# Hereditary angioedema plasma proteomics following specific plasma kallikrein inhibition with lanadelumab

**DOI:** 10.3389/fimmu.2024.1471168

**Published:** 2025-05-09

**Authors:** Dan Sexton, Anton Kichev, Salomé Juethner, Dave Yeung, Amanda MacDonald, Ezequiel Anokian, Bin Li

**Affiliations:** ^1^ Takeda Development Center Americas, Inc., Cambridge, MA, United States; ^2^ Clarivate PLC, Barcelona, Spain; ^3^ Takeda Pharmaceuticals USA Inc., Lexington, MA, United States

**Keywords:** kallikrein-kinin system, lanadelumab, bradykinin, antibody inhibitor of protease, hereditary angioedema

## Abstract

**Introduction:**

Plasma proteomics analyses were performed to identify novel disease state biomarkers of hereditary angioedema due to C1 inhibitor deficiency (HAE-C1INH) and investigate the biological consequences of specific plasma kallikrein inhibition with lanadelumab.

**Methods:**

Affinity proteomic analyses were performed using plasma from healthy controls (*n*=30) and patients with HAE-C1INH before (baseline, *n*=125) and after 6 months of treatment with lanadelumab (300 mg every 2 weeks, *n*=112) using the SomaScan platform.

**Results:**

Relative plasma levels for several proteins differed significantly between controls and patients with HAE-C1INH, and between matched baseline and post-treatment samples from patients with HAE-C1INH. As expected, C1 inhibitor and complement C4 were significantly lower (*P*<1.10e-39 false discovery rate [fdr], *P*<6.6e-25 fdr, respectively) in HAE-C1INH baseline plasma versus controls. Cleaved high-molecular-weight kininogen, a biomarker of excess kallikrein-kinin system (KKS) activation, was higher in HAE-C1INH baseline plasma versus controls (*P*<6.7e-6 fdr) and was reduced in HAE-C1INH plasma after lanadelumab treatment. Of 1041 identified proteins that differed significantly (*P*<0.05) from controls and HAE-C1INH baseline plasma, 120 proteins were no longer different between controls and patients with HAE-C1INH after 6 months of lanadelumab treatment. Canonical pathway and local network analyses of HAE-C1INH plasma proteomics suggest dysregulation in KKS, coagulation, cell adhesion, and connective tissue degradation that approach that of healthy controls following treatment with lanadelumab.

**Conclusion:**

Proteomic analyses of plasma from patients with HAE-C1INH before and after treatment with lanadelumab compared with healthy controls confirmed known HAE-C1INH biomarkers and identified additional potential biomarkers of plasma kallikrein dysregulation for further investigation.

## Introduction

Hereditary angioedema due to C1INH deficiency (HAE-C1INH) is an autosomal dominant genetic disease mediated by a dysregulated plasma kallikrein-kinin system (KKS), which generates excess bradykinin in the vascular compartment; pathophysiology that causes episodic attacks of angioedema (1). KKS activation occurs upon FXII activation to FXIIa, which converts prekallikrein to active plasma kallikrein (PKa), that activates additional FXII and cleaves high-molecular-weight kininogen (HK) to generate cleaved HK (HKa) and bradykinin (1, 2).

Despite the well described pathophysiological role of the KKS and the multitude of approved treatments for HAE-C1INH, the search for novel biomarkers (3) remains in order to elucidate further biological consequences of excess KKS activation, develop improved therapies, and reduce diagnosis time for patients, which for many patients can take 4–9 years on average (4), if the identified biomarkers can be developed as diagnostic assays.

Lanadelumab (Takhzyro, TAK-743, SHP643, DX-2930) is a specific antibody inhibitor of PKa approved to prevent attacks in patients with HAE (5). We performed plasma proteomics with samples from HAE-C1INH patients before and after treatment with lanadelumab to assess the impact of specific PKa inhibition on the plasma proteome.

## Methods

Plasma was collected from patients with HAE-C1INH enrolled in the phase III HELP study for lanadelumab as well as from patients who only enrolled into the open-label extension portion of the HELP study (ClinicalTrials.gov ID: NCT02586805 and NCT02741596, respectively). Plasma was collected from age- and gender-matched healthy controls (*n* = 30) by BioIVT (Westbury, NY). All participants provided written informed consent for blood samples to be used for the investigation of exploratory biomarkers of contact system activation. To minimize ex vivo activation of the contact pathway system during blood collection, plasma was collected from patients with HAE-C1INH and healthy controls by means of a clean venipuncture with a butterfly needle/catheter kit (BD Biosciences part number 367296, San Jose, CA, USA) and removal of the tourniquet upon blood flow to decrease stasis. The first tube of blood was discarded and blood was collected into polypropylene evacuated tubes containing 3.2% sodium citrate (BD Biosciences). Blood samples were centrifuged within 1 hour, and plasma was aliquoted and stored at –80°C until processing.

Plasma proteomic analyses were performed using a multiplex approach that compares relative levels of >7000 proteins (SomaScan, Somalogic, Denver, CO, USA) (6) on the following 3 groups: 1) healthy control plasma (*n* = 30); 2) HAE-C1INH plasma before lanadelumab treatment (baseline, *n* = 125); and 3) HAE-C1INH plasma after 26 weeks of lanadelumab treatment (300 mg Q2W, after treatment, *n* = 114). Of the 114 HAE-C1INH patients for which baseline plasma was available, matched post-treatment samples were available for 112 patients.

## Results

**Figure 1:**
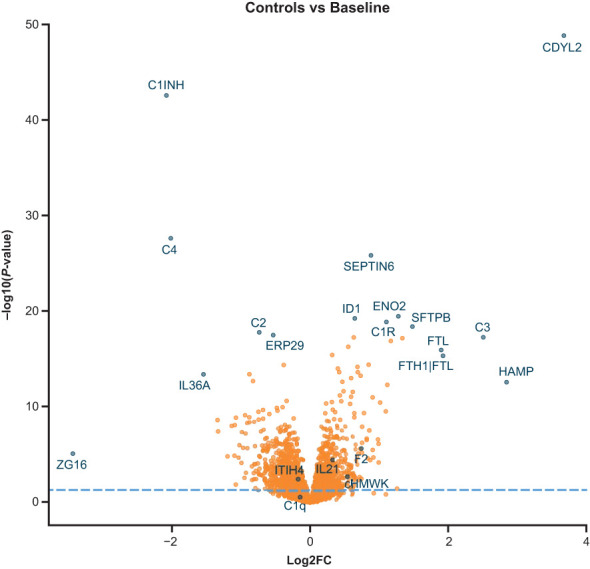
Volcano plot comparison of protein levels from SomaScan analyses in plasma from patients with HAE-C1INH that was collected at baseline to levels present in plasma from age- and gender-matched healthy controls. The x-axis is the fold change (log2FC) in signal for each protein in HAE-C1INH plasma as compared to healthy control plasma. The y-axis is the calculated *P*-value (Log10) for the difference in signal for each protein between HAE-C1INH patients and healthy controls plasma. HAE-C1INH, hereditary angioedema due to C1 inhibitor deficiency.

We compared plasma proteomics of HAE-C1INH plasma at baseline (i.e., before treatment with lanadelumab) to that of healthy control plasma in a volcano plot of *P*-value versus fold change (**Figure 1**). Out of the proteins detected, 1041 proteins were statistically different between HAE-C1INH and healthy controls (**Supplementary Table 1**).

**Figure 2:**
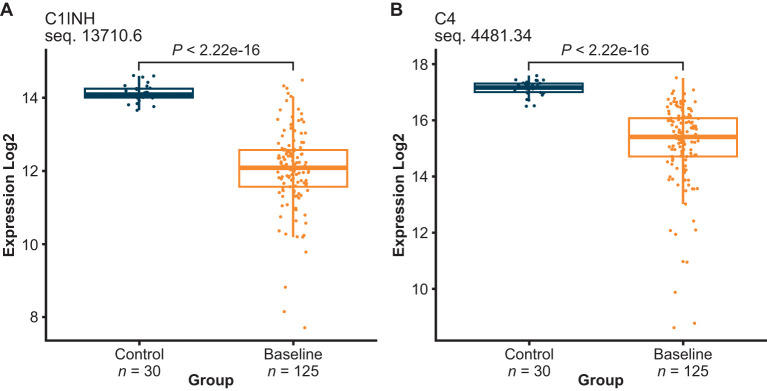
C1INH **(A)** and complement 4 (C4) **(B)** plasma levels were both decreased in HAE-C1INH samples collected at baseline as compared to healthy control plasma. HAE-C1INH, hereditary angioedema due to C1 inhibitor deficiency.

Of the 1041 proteins, C4 and C1INH were lower in HAE-C1INH baseline plasma than healthy control plasma (**Figure 2**), which was expected since low levels of both proteins are used in the diagnosis of HAE-C1INH (7). HAE-C1INH may be associated with C1INH plasma levels that are <50% of a healthy control standard at 1 U/mL (~0.24 g/L) and C4 plasma concentrations below that of healthy controls (8, 9).

We also compared plasma proteomics between healthy controls, baseline HAE-C1INH, and HAE-C1INH after 26 weeks of lanadelumab treatment. Out of the 1041 proteins that were different between healthy controls and HAE-C1INH baseline, 120 proteins were observed to be no longer different from healthy controls after HAE-C1INH patients were treated with lanadelumab for 26 weeks. The list of the 120 proteins is provided in **Supplementary Table 1** and includes HKa, thrombin, tissue kallikrein 14, interleukin-21, α-2-macroglobulin (A2M), and apolipoprotein B (**Supplementary Figure 1**).

**Figure 3:**
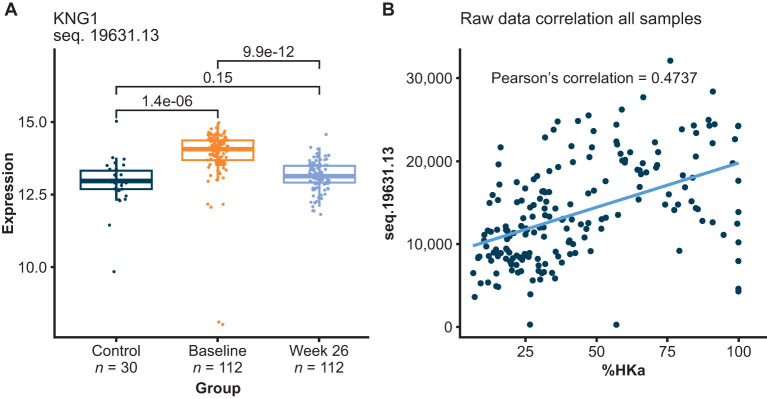
HKa relative levels measured using SOMAmer 19631-13 increased in plasma from HAE-C1INH patients prior to receiving lanadelumab (baseline), relative to healthy controls (Control) and HKa decreased following lanadelumab treatment **(A)**. Correlation between HKa levels as measured using SOMAmer 19631-13 and Western blot analyses of HK **(B)**. SOMAmer 19631-13 was reported by the manufacturer to be raised against kininostatin (domain 5 of HK) with binding observed to HKa (although with~10-folder weaker affinity than for kininostatin) and no binding observed with intact HK or intact LK. HAE-C1INH, hereditary angioedema due to C1 inhibitor deficiency; HK, high-molecular-weight kininogen; HKa, cleaved HK; LK, low-molecular-weight kininogen.

The first one of these 120 potential biomarkers to highlight is HKa, a protein previously shown to be elevated in plasma of patients with HAE-C1INH and a pharmacodynamic biomarker of lanadelumab bioactivity (10–12). **Figure 3** shows the HKa signal measured using SOMAmer sequence 19631-13, which shows that HKa levels were elevated in HAE-C1INH baseline plasma, but were no longer different from healthy control plasma levels after HAE-C1INH patients received lanadelumab for 26 weeks. HKa levels measured using sequence 19631-13 positively correlated to the %HKa measured by Western blot analyses performed during the clinical study with lanadelumab (13). The SomaScan panel contains 3 other SOMAmers against kininogen (15343-337, 7784-1, and 4918-21). The profile for 15343-337 appeared similar to 19631-13, whereas the profile of 7784-1 suggests preferential binding to intact HK, and the profile of 4918-21 suggests that it may bind both HK and HKa (**Supplementary Figures 2**, **3**). Furthermore, the observed reduction in HKa signal using SOMAmers 19631-13 and 15343-337 in paired HAE-C1INH patient samples collected at baseline and after 26 weeks of lanadelumab treatment is consistent with the pharmacodynamic activity previously reported for this PKa inhibitor (**Supplementary Figure 4**) (14).

We next investigated whether additional potential biomarkers from the 120 proteins discovered previously would show measurable protein differences with commercially available ELISA kits. To test this, additional plasma samples (from different HAE-C1INH patients from the same clinical study with lanadelumab and different healthy controls) were analyzed using commercially available ELISA kits (RayBiotech, Peachtree Corners, GA) for interleukin-21, A2M, and apolipoprotein B. We observed no differences between healthy controls and HAE-C1INH plasma for these 3 proteins (data not shown). Differences between results obtained using affinity proteomic methods, including SomaScan, and immunoassays have been previously reported and potentially attributed to differences in the epitopes or protein complexes targeted between the different assays (15). Furthermore, targeted immunoassays often disagree with each other, so disagreement with a proteomic platform does not definitively imply which result is superior. Consequently, the lack of concordance between methods does not demonstrate that the results from the affinity proteomic analyses are necessarily incorrect and require further validation (e.g., using assays based on the specific SOMAmers used for each protein).

We next performed pathway enrichment analysis on these 120 altered proteins using Metacore (Clarivate). As shown in **Table 1**, the KKS is the most significant enriched pathway, which is consistent with the fact that this is a study on patients with HAE-C1INH and the effects of their treatment. Other enriched pathways include blood coagulation, cell adhesion, and connective tissue degradation. These 120 proteins were then analyzed using a known knowledge network approach called Causal-ASsociational NETwork (CASNET), which refers to consistently active subnetworks of known protein-protein interactions (16). Finding active subnetworks in diseases can be a way of generating biological insights, including novel feedback loops, using data with differentially expressed genes or in our case different protein expression levels from proteomic analyses (**Supplementary Figure 5**). Proteins identified by CASnet analyses out of the list of 120 proteins include proteases (thrombin, tissue kallikreins, cathepsin K, plasminogen) protease inhibitors (inter-alpha trypsin inhibitor heavy chain 4, A2M), apolipoproteins, and complement system proteins (**Supplementary Table 2**, **Supplementary Figure 5**).

**Table 1:** Pathway analysis using the 120 proteins.

Metabase process network pathway^13^	r	R	n	N	Z-score	*P*-value	q-value
Kallikrein-kinin system	13	68	187	7113	8.538585	0	3e-06
Blood coagulation	5	68	93	7113	4.409437	0.00186	0.138223
Cell adhesion: Amyloid proteins	7	68	197	7113	3.799111	0.00260	0.138223
Cell adhesion: Platelet-endothelium-leukocyte interactions	6	68	174	7113	3.420398	0.00613	0.243986
Proteolysis: Connective tissue degradation	4	68	118	7113	2.73963	0.02597	0.707345

Where r: intersection of proteomic experiment with ontology term in Metabase; R: size of user’s experiment; n: size of map/process; N: size of “background list” - total number of network objects in ontology; Z-score: enrichment Z-score (the more Z-score, the more significant enrichment); *P*-value: enrichment *P*-value from hypergeometric test; q-value: false-discovery rate–adjusted *P*-value from hypergeometric test.

## Discussion

Biomarker discovery in HAE-C1INH remains an active area of research that can provide novel insights into pathophysiology and may identify novel biomarkers to potentially improve diagnosis (3, 17–20). We used an affinity proteomic platform capable of measuring relative amounts of more than 7000 different human proteins to compare the plasma proteome of patients with HAE-C1INH to that of healthy controls. In addition, we investigated the effect of PKa inhibition using a highly specific antibody inhibitor (lanadelumab) on the plasma proteome of patients with HAE-C1INH.

SomaScan proteomics includes the known diagnostic biomarkers of HAE-C1INH, C1INH and C4, both of which were lower than healthy controls as expected. In addition, SomaScan proteomics measures HKa, a previously identified disease state and pharmacodynamic biomarker of KKS activation (10–12). C1INH, C4, and HKa levels in HAE-C1INH plasma measured using SomaScan proteomics indicate that this technology could be considered for investigations of HAE-C1INH plasma biomarkers and possibly as a pharmacodynamic assay for the investigation of novel therapies targeting the KKS.

By comparing the HAE-C1INH plasma proteome before and after 26 weeks of treatment with lanadelumab to that of healthy controls, we were able to investigate the effect of specific PKa inhibition on the 1041 proteins with levels that differed between HAE-C1INH plasma baseline and healthy controls. These 1041 proteins are listed in the **Supplementary Table 1** and may find use in further studies into the biological consequences of excess KKS activation in HAE-C1INH and other diseases potentially mediated by the KKS, including comorbidities associated with HAE-C1INH (21–23). From this analysis, we identified 120 out of the 1041 proteins that were no longer different from that of healthy controls after 26 weeks of lanadelumab treatment.

A2M, one of these 120 proteins, is a broad-spectrum covalent protein inhibitor of proteases, including PKa, which has multiple functions, including binding and regulating pro-inflammatory cytokines and hormones (24). A2M has been investigated as a biomarker for a number of different diseases, including diabetes mellitus (25, 26). We observed that A2M was elevated in HAE-C1INH plasma relative to that of healthy controls and returned to approximate healthy control levels after 26 weeks of treatment with lanadelumab (**Supplementary Figure 1**). Further studies could elucidate whether the SOMAmer against A2M also binds the A2M-PKa covalent complex, which has been shown to be elevated during an attack in plasma from patients with HAE-C1INH (27).

Complement protein C3 is another one of 120 altered proteins that has been implicated in the pathophysiology of many diseases including diabetic macular edema (28). C3 is a central protein component of the complement system (29). The observation here of increased C3 cleavage fragments (C3b, C3a, and C3d) in baseline HAE-C1INH plasma as compared with that of healthy controls, followed by a reduction upon lanadelumab treatment is consistent with previous reports of C3 activation by PKa (**Supplementary Figure 6**) (30).

Apolipoprotein B, a protein involved in cholesterol deposition in the arterial wall and a marker of cardiovascular risk (31), was among the 120 altered proteins that were elevated in HAE-C1INH baseline plasma and reduced after 26 weeks of treatment with lanadelumab to levels comparable with that of healthy controls. It was previously reported that apolipoprotein B is cleaved by PKa (32). Consequently, it could be useful in future studies to determine whether the SOMAmer against apolipoprotein B exhibits differential binding to intact protein compared with the PKa-cleaved protein. Further investigation into a role for PKa in cholesterol metabolism is supported by the recent observation that prekallikrein binds the low-density lipoprotein receptor and could be a therapeutic target to decrease cholesterol (33).

Another protein among the 120 altered proteins worthy of further investigation is interleukin-21, which is a pleiotropic cytokine involved in T helper 17 cell expansion that was previously identified as being elevated in patients with HAE (34). Inflammation and elevated cytokine levels have been suggested as potentially contributing to attack onset triggers (35).

The pathways identified from comparing pre-dose HAE-C1INH plasma proteomics to that of healthy controls are supported by previous observations. For example, biomarkers of blood coagulation and fibrinolysis have previously been shown to be associated with HAE-C1INH, especially upon attack onset (36). Even though patients with HAE-C1INH may not have an increased risk of thrombosis (37), recent preclinical and clinical studies indicate that high plasma levels of C1 INH reduce the risk of venous thromboembolism (38). Pathway analyses using this proteomic data also identified differences in cell adhesion pathways based on plasma proteomics in HAE-C1INH that appeared to approach levels in healthy controls after treatment with lanadelumab. Elevated levels of adhesion proteins such as VE-cadherin have also been previously shown in plasma from HAE-C1INH patients (39, 40).

In summary, we used plasma proteomic analyses to identify potential HAE disease state biomarkers for further examination with additional patient samples and orthogonal methods for confirmation. Combining proteomic analyses with matched samples from patients before and after treatment with a highly specific antibody therapeutic can help identify active subnetworks in the disease that are mediated by the target.

## Data Availability

The original contributions presented in the study are included in the article/**Supplementary Material**. Further inquiries can be directed to the corresponding author.
