# Supplementary material for: Hereditary angioedema plasma proteomics following specific plasma kallikrein inhibition with lanadelumab
Source: Front Immunol. 2025 May 9;15:1471168. doi: 10.3389/fimmu.2024.1471168 (PMC12098075; doi:10.3389/fimmu.2024.1471168)
Supplement: Supplementary file 1 [file DataSheet1.docx]

**Supplementary Material**

**Supplementary Table 1.** List of 1041 proteins that differed in HAE-C1INH from healthy control plasma and the 120 that no longer differed following lanadelumab treatment. HAE-C1INH, hereditary angioedema due to C1 inhibitor deficiency.

**Supplementary Table 2.** List of proteins from local network analyses in Supplementary Figure 5*

| **Abbreviations** | **Proteins** |
| --- | --- |
| A2M | α-2-macroglobulin |
| ALB | Albumin |
| ADIPOQ | Adiponectin |
| APOB | Apolipoprotein B |
| APOD | Apolipoprotein D |
| C3a, b, and d | Complement 3 fragments |
| C1QTNF9 | Complement C1q and tumor necrosis factor-related protein 9A |
| CTSK | Cathepsin K |
| F2 | Thrombin |
| FABP1 | Fatty acid-binding protein, intestinal |
| FGG | Fibrinogen gamma chain |
| ITIH4 | Inter-α-trypsin inhibitor heavy chain H4 (aka sgp120) |
| KLK3 | Tissue kallikrein 3 |
| KLK13 | Tissue kallikrein 13 |
| KLK14 | Tissue kallikrein 14 |
| KNG1 | Kininogen |
| PLG | Plasminogen |
| PRMT1 | Protein arginine N-methyltransferase 1 |
| SAR1B | GTP-binding protein SAR1b |

* Proteins in red were elevated, whereas those in blue were decreased when hereditary angioedema baseline was compared with that of healthy control plasma. The proteins in gray were added to the active subnetwork via known pathway associations.

**
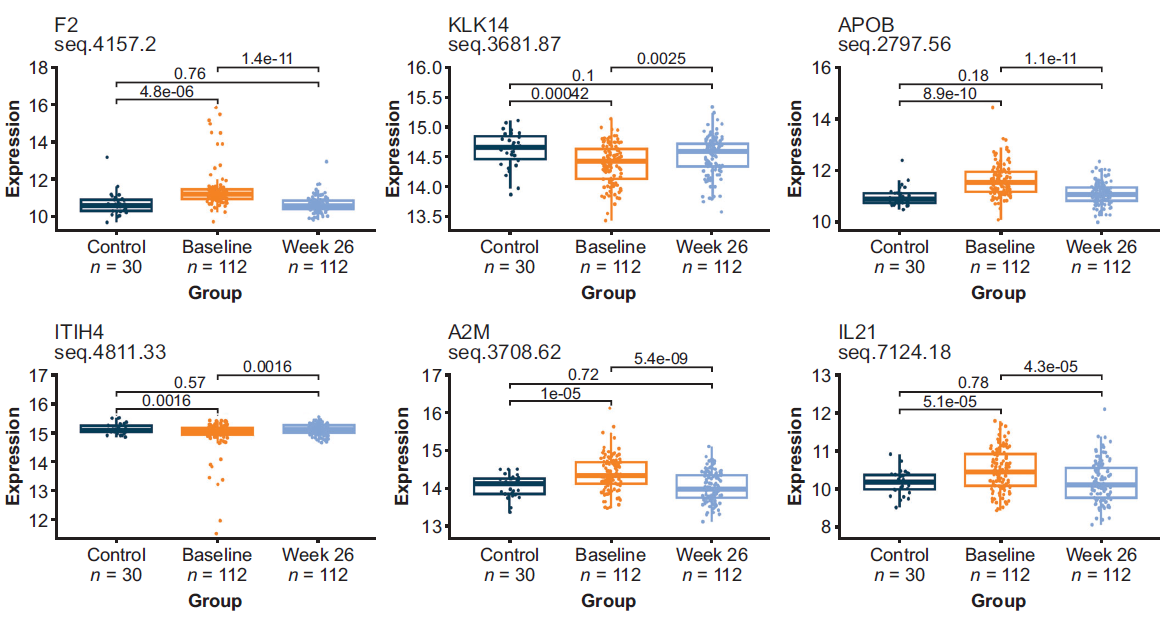
Supplementary Figure 1.** Selected proteins out of the list of 120 proteins that were different between healthy controls and HAE-C1INH baseline plasma and approached that of healthy control levels following treatment with lanadelumab. APOB, apolipoprotein B; A2M, α-2-macroglobulin; F2, thrombin; HAE-C1INH, hereditary angioedema due to C1 inhibitor deficiency; IL-21, interleukin-21; ITIH4, inter-α-trypsin inhibitor heavy chain H4 (aka sgp120); KLK14, tissue kallikrein 14.


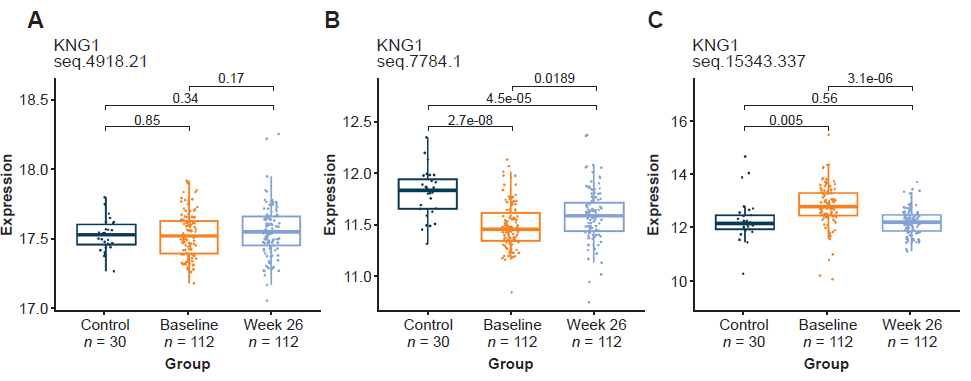


**Supplementary Figure 2.** Relative signals observed for 2 SOMAmers listed in the SomaScan panel as binding to kininogen (KNG1). SOMAmer 4918-21, reported by the manufacturer (Somalogic) to be raised against intact high-molecular-weight kininogen (HK), appears in this analysis not to bind differentially to intact (HK) versus cleaved kininogen (HKa) (A). The signal observed for SOMAmer 7784-1 appears consistent with the report by the manufacturer as being selective for intact HK with some binding to HKa and low-molecular-weight kininogen (LK) that was at least 10-fold weaker affinity than intact HK (B). SOMAmer 15343-337 was reported to be specific for HKa with no binding observed with intact HK or intact LK (C).


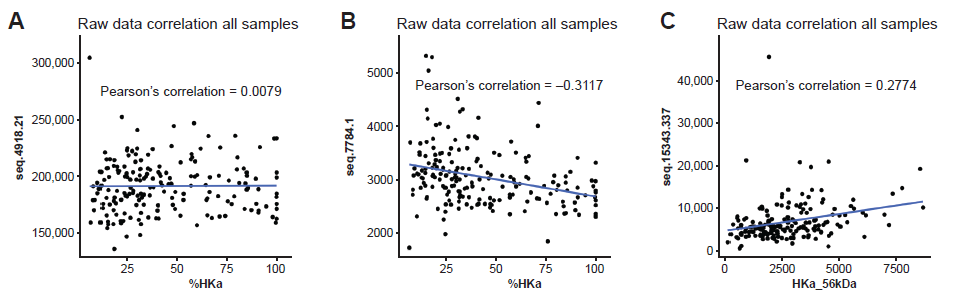


**Supplementary Figure 3.** Correlations between SomaScan signal towards HKa or HK and Western blot analyses. No correlation between SomaScan signal with SOMAmer 4918.21 and the %HKa measured by Western blot analyses, which suggests that it may bind both HK and HKa (A). The inverse correlation observed between SOMAmer 7784.1 and the %HKa measured by Western blot analyses suggests that this SOMAmer may bind preferentially to HK (B). The positive correlation observed between SOMAmer 15343.337 and the %HKa measured by Western blot analyses suggests that this SOMAmer may bind preferentially to HKa (C). HK, high-molecular-weight kininogen; HKa, cleaved HK.


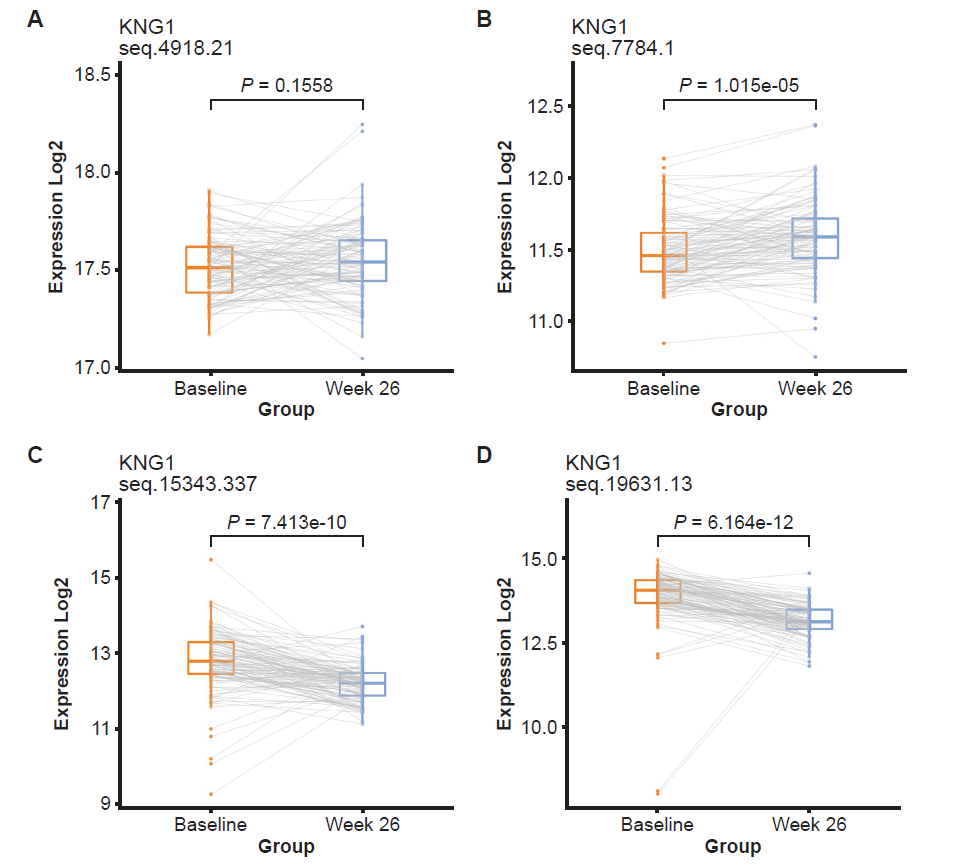


**Supplementary Figure 4.** SomaScan signal for HK/HKa in HAE-C1INH plasma before (baseline) and after 6 months of treatment with lanadelumab (Week 26). The signal with SOMAmer 4918.21 (A) was not affected by lanadelumab treatment, suggesting that it binds cleaved and intact kininogen. The mean signal with SOMAmer 7784.1 increased with lanadelumab treatment, suggesting that it preferentially binds intact HK (B). The signal with SOMAmers 15343.337 (C), and 19631.13 (D) decreased in HAE-C1INH patients’ plasma following lanadelumab treatments, indicating that these SOMAmers are selective for HKa. HAE-C1INH, hereditary angioedema due to C1 inhibitor deficiency; HK, high-molecular-weight kininogen; HKa, cleaved HK; KNG1, kininogen; LK, low-molecular-weight kininogen.


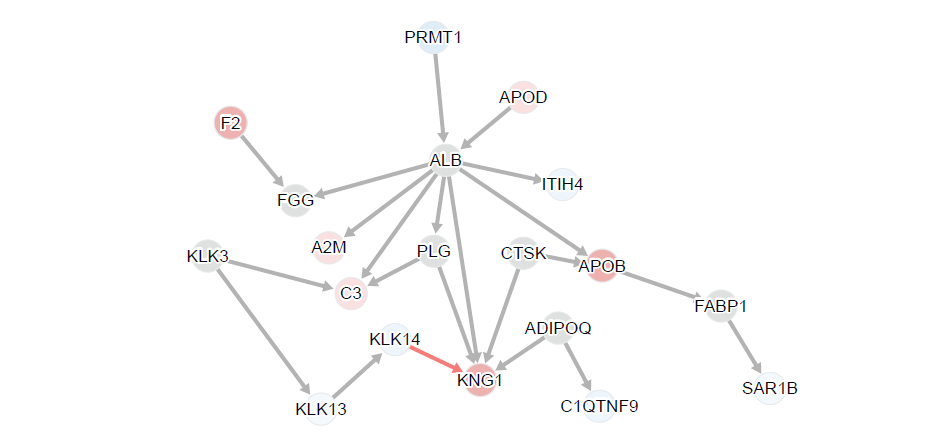


**Supplementary Figure 5.** Identified proteins (see Supplementary Table 2) incorporated into known knowledge network, CASnet incorporates the directionality of the change in protein level, where proteins in red were elevated, whereas those in blue were decreased when HAE baseline was compared with healthy control plasma (Supplementary Figure 6). The proteins in gray were added to the active subnetwork via known pathway associations. HAE, hereditary angioedema.


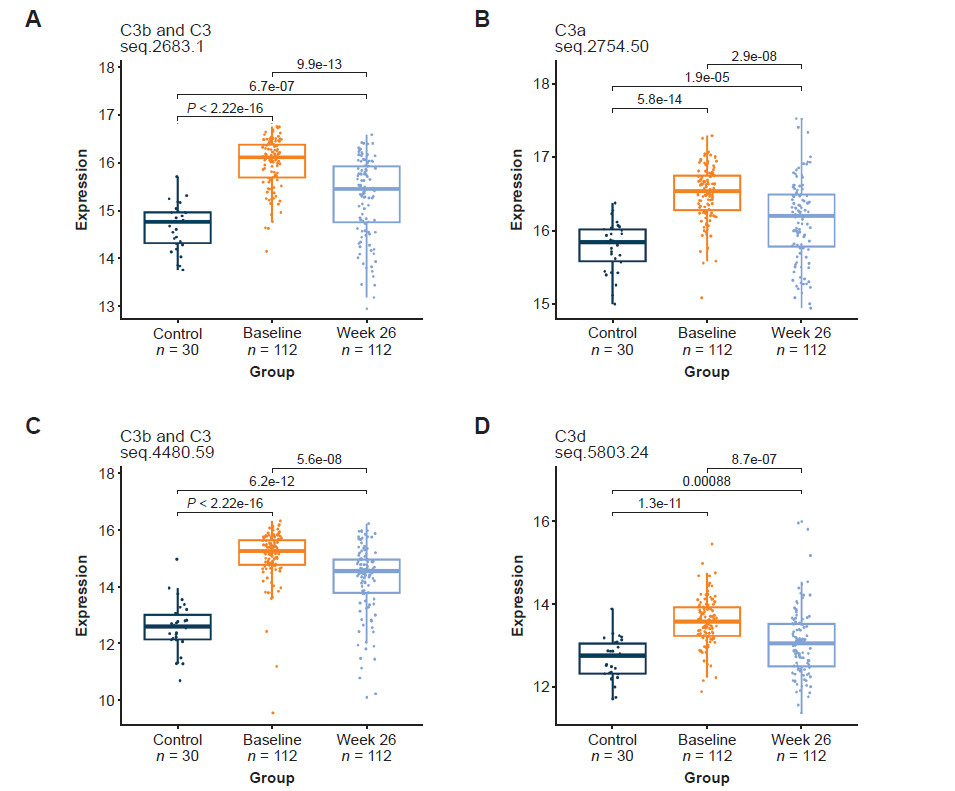


**Supplementary Figure 6.** Complement C3 fragments were elevated in HAE-C1INH plasma relative to healthy control plasma but no longer different following lanadelumab treatment. SOMAmer 2683.1 was reported by the manufacturer to bind C3 and C3b with similar affinity (A). SOMAmer 2754.50 was reported to be selective for C3a and C3a-des-Arginine (B). SOMAmer 4480.59 was reported to be selective for binding to C3 and C3b (C). SOMAmer 5803.24 was reported to be selective for binding to C3d (C). HAE-C1INH, hereditary angioedema due to C1 inhibitor deficiency; HK, high-molecular-weight kininogen.
